# Co‐occurrence of progressive hemifacial atrophy due to morphea with homolateral segmental vitiligo: A case report

**DOI:** 10.1002/ccr3.4458

**Published:** 2021-07-21

**Authors:** Pukar Chapagain, Sudha Agrawal

**Affiliations:** ^1^ Department of Dermatology and Venereology BP Koirala Institute of Health Sciences Dharan Nepal

**Keywords:** hemifacial atrophy, segmental vitiligo

## Abstract

Simultaneous occurrence of progressive hemifacial atrophy due to morphea and homolateral segmental vitiligo, younger onset, rapid progression followed by stabilization and dermatomal distribution suggests a possible relationship between them.

## Graphical abstract

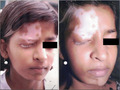

## 1 INTRODUCTION

A female presented with segmental vitiligo on the right ophthalmic (V1) nerve distribution followed by hemifacial atrophy on the right mandibular (V3) nerve distribution which stabilized after the treatment with chloroquine and betamethasone pulse. Both dermatoses have younger onset, rapid progression followed by stabilization and dermatomal distribution, suggesting a possible common etiological link.

Progressive hemifacial atrophy is a unilateral, slowly progressive, atrophic disorder of skin and underlying connective tissue.^1^ Segmental vitiligo is an acquired idiopathic condition of localized depigmentation that occurs in the unilateral dermatomal distribution.^2^ Both conditions are distinct entities with autoimmunity and disorder of peripheral nervous system implicated in their pathogenesis, and only a few reports of their simultaneous occurrence have been reported.

## 2 CASE REPORT

A 20‐year‐old female patient presented to Department of Dermatology and Venerology, BP Koirala Institute of Health Sciences with the appearance of depigmented macule on the right half of the forehead and upper eyelid in the distribution of ophthalmic (V1) nerve with leukotrichia including right eyebrow, eyelashes, and frontal scalp hair and a hyperpigmented, atrophied, and indurated lesion on the right half of the chin along mandibular (V3) distribution. The patient first noticed depigmentation of skin at the age of 7, followed few months later by hyperpigmentation on the right half of chin which gradually progressed over a period of 4 years to form atrophic and indurated plaque with deviation of mouth and nose toward the affected side. There was no history of trauma or injury or vaccination to the site prior to onset of lesion, family history of similar lesions, diminished vision headache, seizures, or difficulty in opening mouth.

**FIGURE 1:**
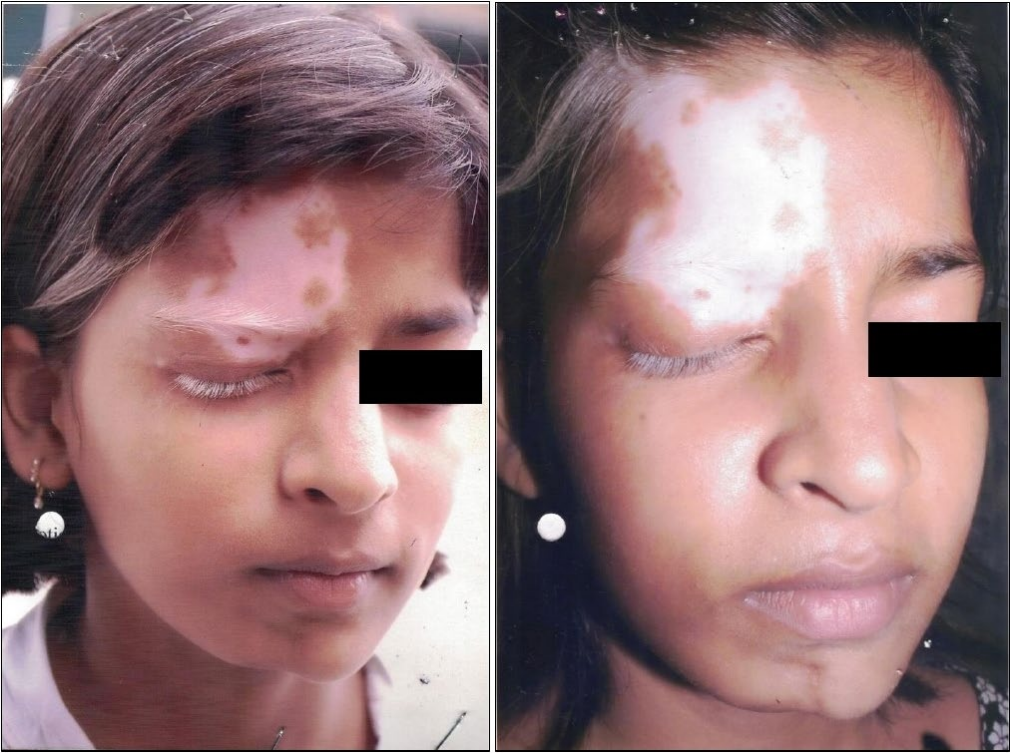
(A) and (B) Segmental vitiligo on the right V1 nerve distribution (forehead) and atrophic plaque of morphea on the right V3 distribution (chin)

On examination, a well‐defined depigmented macule of size 4 cm × 3 cm was present on the right half of forehead and right upper eyelid with leukotrichia including frontal scalp hair, right eyebrow, and eyelashes (Figure 1A,B). Similarly, a hyperpigmented indurated atrophic plaque was present on the right half of chin with visible asymmetry toward the right half (Figure 1A,B).

**FIGURE 2:**
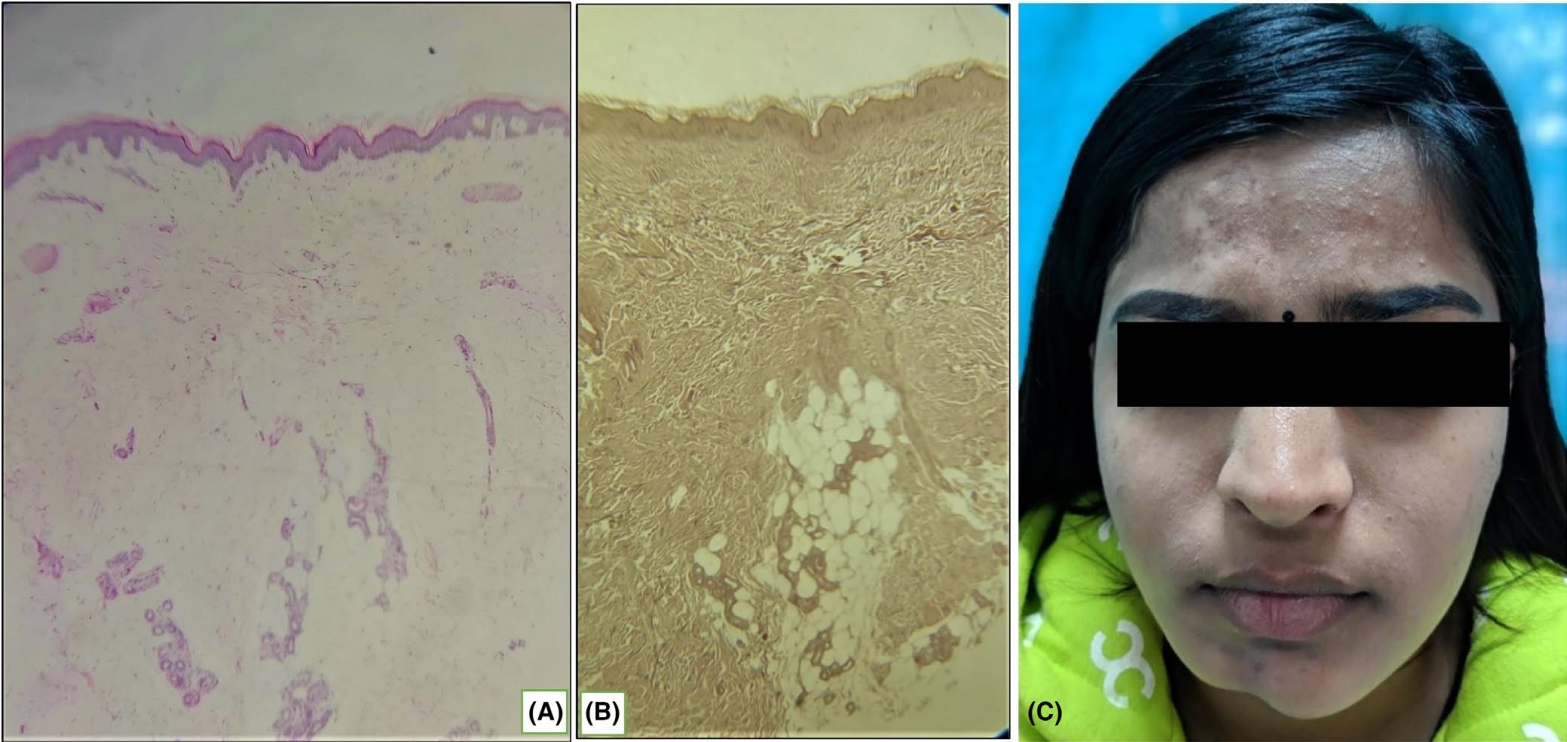
(A) H & E stain of atrophic plaque showing epidermal atrophy, homogenization of dermis, and pulled up appearance of subcutis. (B) Verhoeff‐Van Gieson stain (VVG) showing elastin fibers and pulled up appearance of subcutis. (C) Static condition of segmental vitiligo and hemifacial atrophy 9 years after the treatment

The general physical examination other than cutaneous examination was unremarkable. Laboratory studies including complete blood cell count, erythrocyte sedimentation rate (ESR), thyroid function tests, antinuclear antibody, and urine analysis were all negative or within normal ranges. Radiological features of the skull showed no bony involvement. Skin biopsy from atrophic plaque revealed epidermal atrophy with homogenization of dermis, markedly reduced adnexal structures, and pulled up appearance of subcutis which was consistent with morphea (Figure 2A,B).

She was managed with topical tacrolimus 0.1%, cream fluticasone 0.05%, and oral chloroquine 125 mg three times a day for a duration of 3 months for progressive hemifacial atrophy and topical tacrolimus 0.1%, cream fluticasone 0.05%, topical PUVA, and betamethasone oral mini pulse for vitiligo for 3 months after which the disease progression stopped. The oral treatment was gradually tapered off in 6 months, and topical treatment was maintained for a year. The disease has remained static since last 9 years, and she is planned for surgical correction of the progressive hemifacial atrophy (Figure 2C).

## 3 DISCUSSION

Progressive hemifacial atrophy and segmental vitiligo are two distinct disease entities, which lie within the autoimmune spectrum of disease. The cases of co‐occurrence of progressive hemifacial atrophy and homolateral segmental vitiligo are characterized by an onset at younger age, rapid progression followed by stabilization and dermatomal distribution.^3^, ^4^, ^5^, ^6^, ^7^, ^8^, ^9^, ^10^ Segmental vitiligo is an acquired idiopathic condition of localized depigmentation in a unilateral dermatomal distribution that results from progressive loss of functional melanocytes.^2^ The pathogenesis of the segmental vitiligo is unclear, and various hypotheses have been put forward including sympathetic nerve dysfunction and immune pathomechanism. Different causative factors have been postulated in morphea such as immunological abnormalities, trauma neurological abnormalities, and infectious diseases.^3^, ^11^ There were no significant family history, history of trauma, or infections preceding the onset of skin lesion and no any other significant clinical physical findings suggestive of other possible causes for progressive hemifacial atrophy and segmental vitiligo.

## 4 CONCLUSION

The cases of simultaneous occurrence of progressive hemifacial atrophy with homolateral segmental vitiligo are presented at a younger age, progresses rapidly followed by stabilization and more or less dermatomal distribution, suggesting a possible relationship between them although the mere coincidental coexistence cannot be excluded.

## CONFLICT OF INTEREST

None declared.

## AUTHOR CONTRIBUTIONS

PC: prepared, edited, and reviewed the literature. SA: contributed to idea and concept.

## ETHICAL APPROVAL

This case report was ethically approved by Institutional Review Committee (IRC) of BP Koirala Institute of Health Sciences (BPKIHS).

## CONSENT STATEMENT

Patient provided written consent for publication of this case report. It is available upon request.

## Data Availability

Data will be made available upon request.
